# Thermal Expansion of a Multiphase Intermetallic Ti-Al-Nb-Mo Alloy Studied by High-Energy X-ray Diffraction

**DOI:** 10.3390/ma14040727

**Published:** 2021-02-04

**Authors:** Peter Staron, Andreas Stark, Norbert Schell, Petra Spoerk-Erdely, Helmut Clemens

**Affiliations:** 1 Institute of Materials Research, Helmholtz-Zentrum Geesthacht, Max Planck-Straße 1, 21502 Geesthacht, Germany; andreas.stark@hzg.de (A.S.); norbert.schell@hzg.de (N.S.); 2 Department Materials Science, Montanuniversität Leoben, Franz Josef-Straße 18, 8700 Leoben, Austria; petra.spoerk-erdely@unileoben.ac.at (P.S.-E.); helmut.clemens@unileoben.ac.at (H.C.)

**Keywords:** intermetallics, TiAl alloys, thermal expansion, high-energy X-ray diffraction

## Abstract

Intermetallic γ-TiAl-based alloys are lightweight materials for high-temperature applications, e.g., in the aerospace and automotive industries. They can replace much heavier Ni-based alloys at operating temperatures up to 750 °C. Advanced variants of this alloy class enable processing routes that include hot forming. These alloys consist of three relevant crystallographic phases (γ-TiAl, α_2_-Ti_3_Al, β_o_-TiAl) that transform into each other at different temperatures. For thermo-mechanical treatments as well as for adjusting alloy properties required under service conditions, the knowledge of the thermal expansion behavior of these phases is important. Therefore, thermal expansion coefficients were determined for the relevant phases in a Ti-Al-Nb-Mo alloy for temperatures up to 1100 °C using high-energy X-ray diffraction.

## 1. Introduction

Intermetallic titanium aluminide alloys based on the γ-TiAl phase are promising materials for high-temperature applications up to service temperatures of about 750 °C. They have replaced much heavier Ni-based superalloys for low-pressure turbine blades in jet engines [1,2]. Other applications can be found in the automotive industry for racing car engine valves or turbocharger wheels in passenger cars [3]. Various alloy compositions and processing routes are still being studied intensively for further improvements of this material. For reviews on this novel lightweight structural material, see, e.g., [4,5].

Industrial applications of a material require good processing properties. Forging is an essential processing step that shapes many structural materials. The class of β-stabilized TiAl alloys enable hot forging via the good deformability of the β-TiAl phase (cubic body-centered A2 structure) at high temperatures [6]. After the forging process, TiAl alloys are subjected to particular heat treatments in order to obtain balanced mechanical properties as reported in [1,3,5]. Besides the β-phase, which transforms to the ordered β_o_ phase (B2 structure) upon cooling, γ-TiAl alloys are composed of γ-TiAl (tetragonally distorted face-centered cubic L1_0_ structure) and α_2_-Ti_3_Al (hexagonal D0_19_ structure) phases [7]. Each phase can have slightly different heat expansion properties, especially at high temperatures. The knowledge of the coefficients of thermal expansion (CTE) is important for designing industrial heat treatments involving heating and cooling cycles. It is well known that fast cooling of γ-TiAl alloys, e.g., after welding, can lead to cracking due to the build-up of high residual stresses [8].

CTE are mostly determined by measuring the length or volume change of a sample as a function of temperature; different techniques exist for this purpose [9,10]. Little data on the CTE of intermetallic TiAl alloys exist in the literature. He et al. determined CTE for γ-TiAl single crystals in the temperature range of 27–373 °C using a resonant ultrasound spectroscopic technique [11]. Zhang et al. compiled results from the literature for Ti-50Al (in at%), Ti-47Al-4(Nb,W,B), and Ti-47Al-1.5(Nb,Cr,Si) in the temperature range from RT to 600–900 °C [12]. A few tabulated values for single crystals and polycrystalline bulk materials are found in [13].

However, to understand the expansion of the atomic lattice in the polycrystal, diffraction measurements are required. Especially when the material consists of more than one phase, the phase-sensitive diffraction can yield information of all relevant phases. Some early in-situ X-ray diffraction (XRD) studies of TiAl alloys focused more on the phase transformations than on the CTE [14,15]. Liss et al. studied the lattice parameter evolution of Ti-45Al-7.5Nb-0.25C under pressure [16]. Li et al. determined the lattice strain evolution in a Ti-45Al-7.5Nb-(0.25,0.5)C alloy at atmospheric and high pressure using synchrotron X-ray diffraction [17]. Gaitzenauer et al. determined the CTE for the TNM alloy (see next paragraph) [18], which is to our knowledge the only experimental study on CTE in TNM. Additionally, there is only one study, by Holec et al. [19], in which ab-initio calculations of thermal lattice expansion in γ-TiAl and α_2_-Ti_3_Al phases were performed.

In this work, we studied the CTE of the γ, α_2_, and β_o_-phases in the so-called TNM alloy in the temperature range from RT to 1100 °C. The TNM alloy has a nominal composition of Ti-43.5Al-4Nb-1Mo-0.1B (in at%) and represents a 4th generation γ-TiAl-based alloy, i.e., a process-adapted alloy with excellent hot-workability as an outstanding feature, see [20]. XRD was used to determine the lattice parameters as a function of temperature. A synchrotron source delivering high-energy X-rays yielded sufficient penetration to study several-millimeter-thick samples to ensure the results were taken from the bulk of the material. A commercial quenching dilatometer was used in the X-ray beam for a precise temperature control. The results contribute to a comprehensive description of the thermo-physical properties of this alloy class.

## 2. Materials and Methods

The TNM material of the present investigation was produced by GfE Metalle und Materialien GmbH, Nuremberg, Germany, via the so-called “advanced beta process”. By this technique, a powder metallurgically compacted electrode is vacuum arc remelted (VAR) to achieve adequate chemical and structural homogeneity. Subsequently, the electrode is melted in a VAR skull melter and cast by a centrifugal casting process. Detailed information on the applied melting technique can be found in [21]. The ingot had a diameter of 55 mm and a raw length of about 280 mm. The as-cast condition shows a small fraction of residual micropores, which are eliminated by an ensuing hot isostatic pressing (HIP) step. Thereby, the material is heated up to 1200 °C, held for 4 h at 200 MPa, and subsequently cooled via furnace cooling to RT. A part of the ingot is then subjected to hot-forging within the (α + β) phase field region, followed by controlled furnace cooling as described in [22]. From this material, the specimens used in this study were cut out using electrical discharge machining. The chemical composition of the TNM material was Ti-43.05Al-4.05Nb-0.94Mo-0.08B (in at%). As analytical methods, X-ray fluorescence spectroscopy, inductively coupled plasma-optical emission spectroscopy, and carrier gas hot extraction analysis were used.

**Figure 1:**
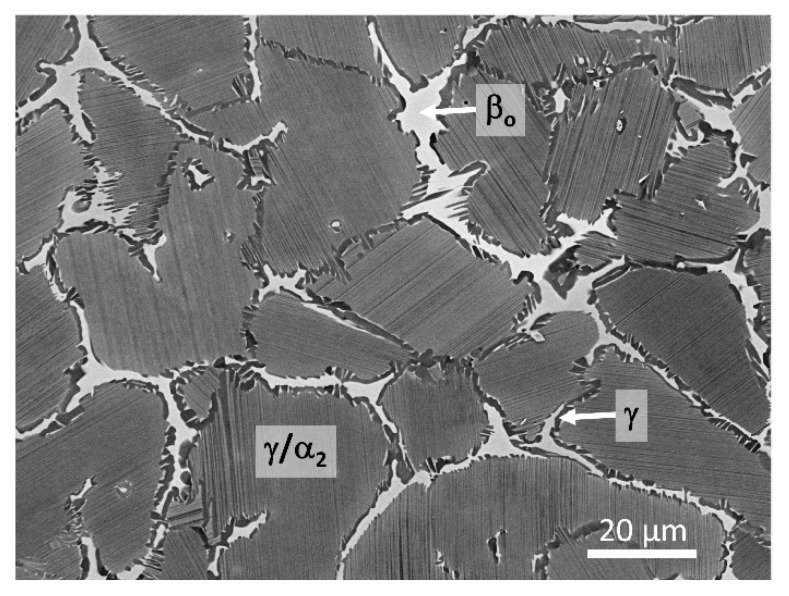
Microstructure of the investigated TNM alloy in the forged condition. The lamellar colonies consist of γ (dark contrast) and α_2_ (light contrast) laths. The surrounding phases are γ and β_o_ (bright contrast). Scanning electron microscope image taken in back-scattered electron mode.

The microstructure of the forged TNM alloy is shown in Figure 1 and consists of lamellar γ/α_2_ colonies surrounded by seams of β_o_ and γ phases. The colony sizes are in the range of 20 to 60 µm. The thickness of the laths within the colonies is within the sub-µm range [22]. In general, the microstructure depicted in Figure 1 is rather homogeneous and thus no significant texture is expected. The evolution of the microstructure of the TNM alloy during solidification, HIP, and subsequent hot-forging/controlled cooling is well understood; for additional detailed information, the reader is referred to the following references [3,22,23]. At this point, it should be mentioned that the results presented in Section 3 show that upon heating up to about 800 °C there are no changes in the phase fractions and with heating up to 1100 °C there are only very small changes. Therefore, no changes in the composition of the constituting phases are expected. Thus, the microstructure can be considered thermally stable in the temperature range where the CTE were determined.

Lattice parameters of the α_2_, γ, and β_o_ phases were determined by means of high-energy X-ray diffraction. The measurements were performed at the beamline P07B (HEMS), operated by HZG, at the PETRA III synchrotron source of DESY, Hamburg [24]. The X-ray energy was 87.1 keV, the corresponding wavelength was 0.1423 Å. The beam cross-section was 1 mm × 1 mm. A Perkin Elmer solid-state flat panel X-ray detector with a pixel size of 200 µm was used for recording diffraction patterns. The samples were heated in a commercial dilatometer (Dilatometer 805 A/D from TA Instruments, Newcastle, DE, USA) placed in the X-ray beam [25]. The dilatometer had been modified by introducing windows for the X-ray beam. Ceramic tubes were used for fixing the sample in the dilatometer. The temperature was controlled using a thermocouple welded onto the sample. This process for temperature measurement together with the precise temperature control of the heating system guaranteed a high precision and repeatability of the measurements. The sample had a length of 10 mm and a diameter of 4 mm. The heating rate was set to 20 K min^–1^. The process took place under an Ar inert gas at a pressure of about 500 mbar. The exposure time was 2 s and an image was taken every 7 s. With the given heating rate, this leads to a temperature uncertainty of 0.67 K and a data point every 2.33 K. The sample was heated to 1450 °C, held for 5 min, and then cooled at the same rate. Data above 1300 °C were not analyzed because of the strong grain growth that led to diffraction rings with just a few spots on them.

The two-dimensional diffraction images were reduced to one-dimensional diffraction patterns using the software FIT2D (version 12.077.i686) [26,27]. Full pattern fitting was done for determining lattice parameters and phase fractions of the three present phases using the package Maud [28]. The relative errors in the lattice parameters resulting from the fits up to 1200 °C were between 1.5 × 10^–5^ and 4.5 × 10^–5^ with the exception of the α_2_ *c* value with a relative error of 9.5 × 10^–5^. These errors are small enough for calculating CTE from a large set of data points within a temperature range. The instrument function for the diffraction set-up was determined with a LaB_6_ powder sample.

The CTE is defined as the relative change in length per temperature interval:

(1) $$e_{l}=\frac{\Delta l}{l\cdot \Delta T}$$

where *l* is the length at the reference temperature. The CTE was calculated from the lattice parameters of each of the three phases. Essentially, it is the derivative of the function *l*(*T*). Since measured data always have noise, the calculation of the derivative also has significant noise. Consequently, we avoided extensive smoothing of the data as well as fitting of spline or other functions as this can create artefacts. Instead, we used a polynomial of second order for approximating the function *l*(*T*) within the relevant temperature interval. The derivative of this parabola is a line describing the expansion rate as a function of temperature. The line gives the essential information about the thermal expansion. However, information that is more detailed requires significantly higher measurement efforts.

For isotropic expansion, the volume expansion coefficient can be calculated from the linear expansion rate by

(2) $$e_{v}=3e_{l}$$

The phase-specific change in the crystal unit cell volume is calculated from the expansion of the axes (*a* and *c*) of the crystal lattice for that phase. For the cubic β_o_ lattice, the unit cell volume is *a*^3^, for the tetragonal γ phase, the unit cell volume is *a*^2^*c*, whereas the unit cell volume is *a*^2^*c*$\sqrt{3}$/2 for the hexagonal α_2_ phase. For obtaining a macroscopic bulk value, a weighted mean value of the volume expansion coefficients of the three phases, derived from the change in the lattice parameter, was calculated where the weights are given by the occurring phase fractions *f_i_*:

(3) $$e_{v}=\frac{1}{\Delta T}\left(e_{v,\gamma }\cdot f_{\gamma }+e_{v,\alpha_{2}}\cdot f_{\alpha_{2}}+e_{v,\beta_{o}}\cdot f_{\beta_{o}}\right)$$

## 3. Results and Discussion

### 3.1. Phase Fractions

**Figure 2:**
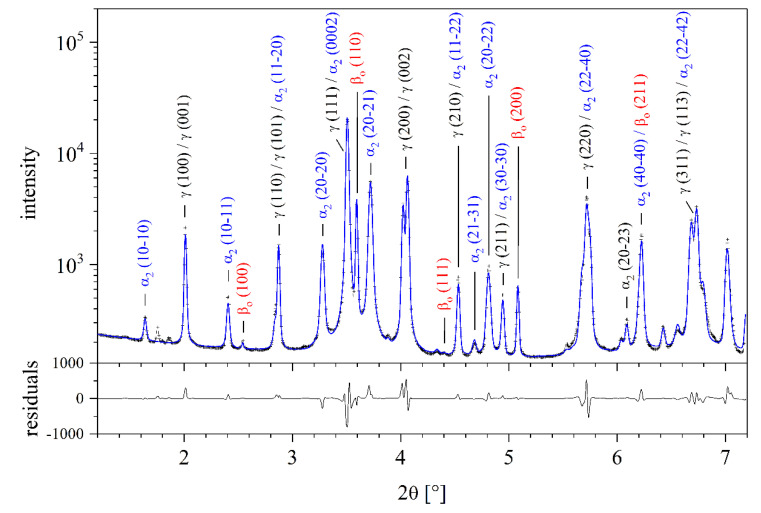
Diffraction pattern of the investigated TNM alloy at RT, measured using X-rays with an energy of 87.1 keV (wavelength 0.1423 Å). The blue line is a fit to the data, calculated with Maud.

The lattice parameters and volume fractions of the three phases present in the forged TNM material were determined by full pattern fitting using Maud [27] (Figure 2). The initial γ, α_2_, and β_o_ phase volume fractions at RT are 69, 24, and 7%, respectively.

**Figure 3:**
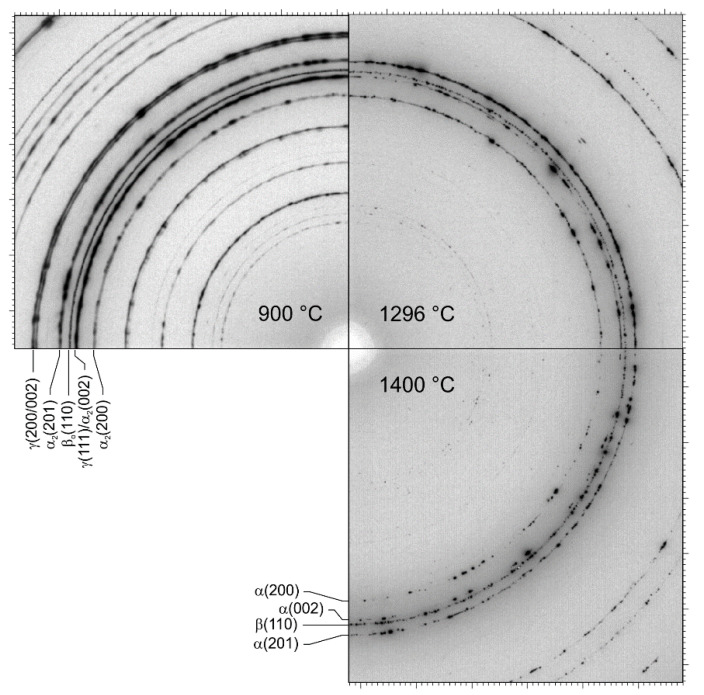
90° sectors of detector images at three different temperatures during heating with 20 K min^–1^ (see text).

The diffraction rings of all phases are inhomogeneous with spots at RT and up to 900 °C (Figure 3). These spots indicate a microstructure with relatively large grains present in addition to smaller grains However, there are no significant intensity variations along the rings on a larger scale, indicating a negligible crystallographic texture. At 1296 °C, the γ phase has transformed and, at this temperature, disordered hexagonal α and bcc β are present. Grain growth is significant and the diffraction rings are not homogeneous, but show distinct intensity spots from large grains.

**Figure 4:**
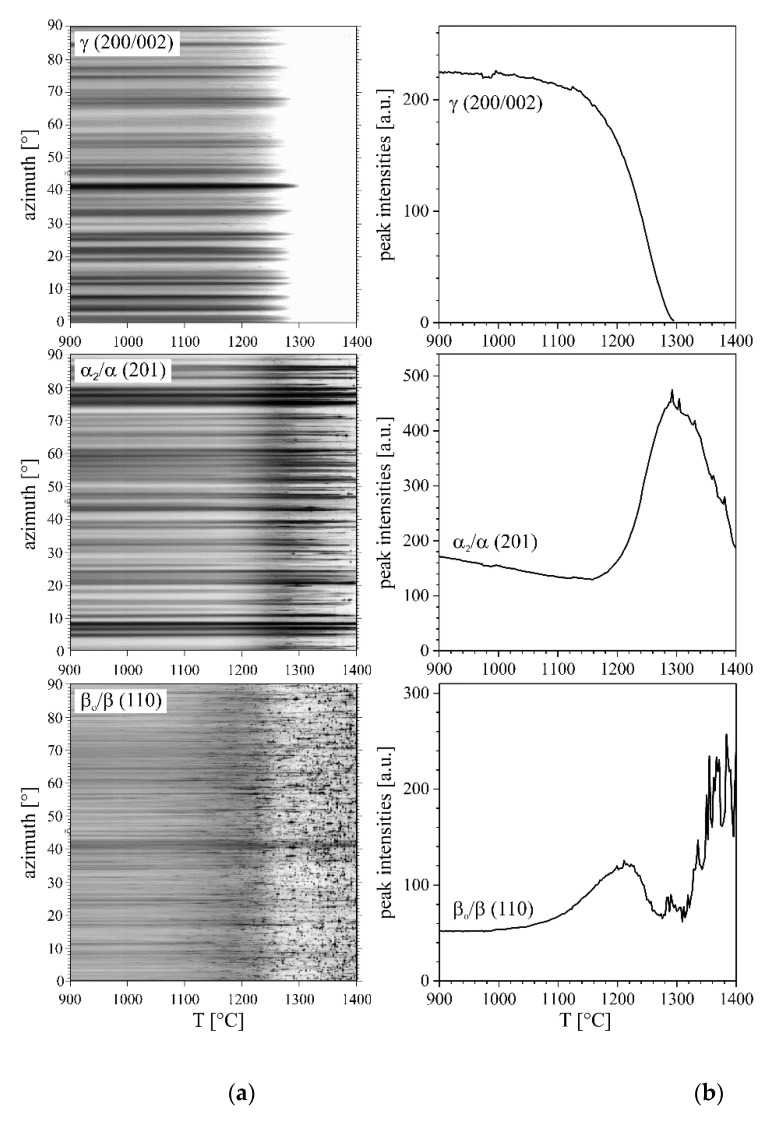
Azimuthal intensity distribution (90° sector) for one reflection of each phase as a function of temperature (**a**) and intensity of the corresponding peak (**b**) during heating with 20 K min^–1^. Grain growth leads to the appearance of spotty intensity patterns.

The development of grain size and texture can be visualized by plotting the intensity of a single peak as a function of azimuth angle and time or temperature. In Figure 4, one relevant peak of each phase is plotted as a function of temperature in an azimuthal range from 0° to 90°. At lower temperatures, the intensity modulations in azimuthal direction originate in large grains. In general, however, the crystallographic texture is weak. Above 1100 °C, the γ phase starts to dissolve without changes in the azimuthal intensity distribution. Thus, grain size and texture of the γ phase do not change during the transformation. The α_2_ phase fraction starts to increase at 1160 °C. Around this temperature, the disordering reaction α_2_ → α takes place. For more information regarding phase transformations (α_2_ → α and β_o_ → β) and phase transformation temperatures in the TNM alloy, the reader is referred to the work of Schmölzer et al. [7]. Grain growth sets in at about 1250 °C. First changes in the β_o_ phase can already be observed at about 1000 °C. At 1150 °C, β_o_ grains start to grow significantly.

**Figure 5:**
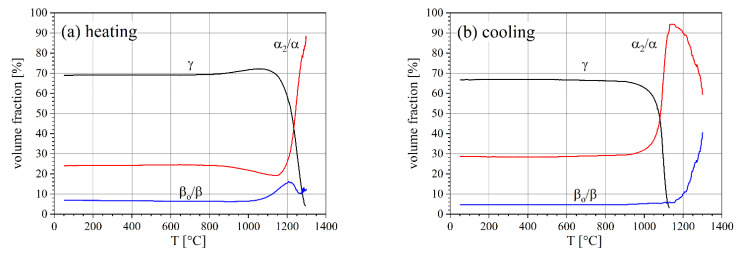
Phase fractions of γ, α_2_/α, and β_o_/β as a function of temperature (**a**) during heating and (**b**) during cooling, both with 20 K min^–1^. The relative error is between 1% and 4% of the volume fractions except for at high temperatures where significant grain growth occurs, leading to larger errors.

These changes correspond to the changes in the phase fractions (Figure 5). The phase fractions are almost constant up to about 800 °C. With increasing temperature, the γ phase starts to dissolve, while the α_2_ content increases. In addition, β_o_ increases slightly up to 1150 °C. Afterwards, a decrease is observed, followed by a recurrent increase. At 1250 °C, the γ phase has almost vanished and the α phase reaches a maximum of 80%. Above 1250 °C, the α phase transforms into β. These observations correspond to other results for this alloy as reported in [7].

A hysteresis is observed during cooling with the same rate (Figure 5). The α phase starts transforming into γ only at about 1125 °C, which is 175 °C below the end of the transformation phase of γ to α during heating. This hysteresis is expected to be due to differences in the chemical composition and grain size of the phases during heating and cooling.

### 3.2. Lattice Parameters

**Figure 6:**
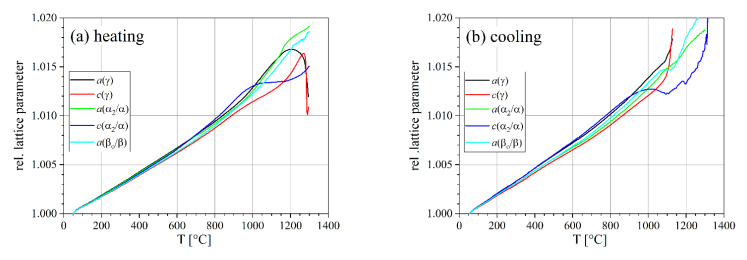
Lattice parameters of the γ, α_2_, and β_o_ phases normalized to the corresponding RT values (**a**) during heating and (**b**) during cooling at a rate of 20 K min^–1^.

For comparison of all lattice parameters, they were normalized to the corresponding value at RT (Table 1). The lattice parameters of the three phases show a very similar behavior up to about 900 °C (Figure 6). Above 900 °C, phase transformation starts slowly and the lattice parameters start to diverge. Heating was carried out up to 1450 °C; however, significant grain growth above 1300 °C made the determination of lattice parameters rather unreliable. Thus, further evaluation was restricted to temperatures up to 1300 °C.

**Figure 7:**
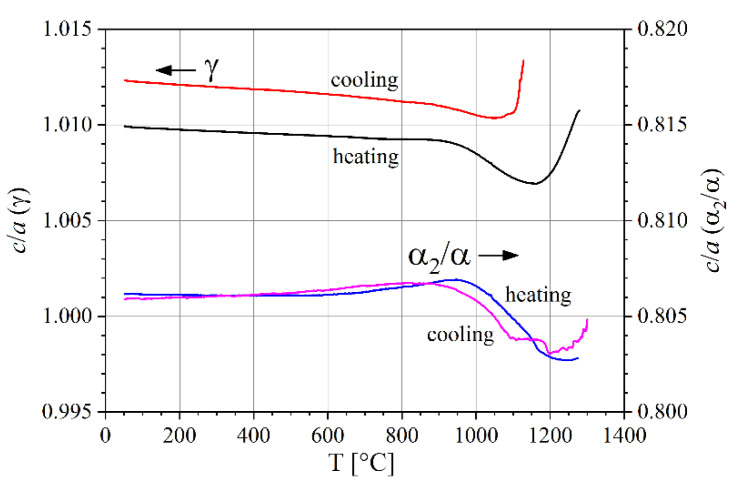
Ratio *c*/*a* of crystal axes for phases α_2_ and γ as a function of temperature.

The ratio *c*/*a* of the crystal axes of the tetragonal γ phase and the hexagonal α_2_ phase are almost constant up to 900 °C. Above 900 °C, they start to decrease. In the case of the γ phase, *c*/*a* increases again above 1150 °C as shown in Figure 7.

**Figure 8:**
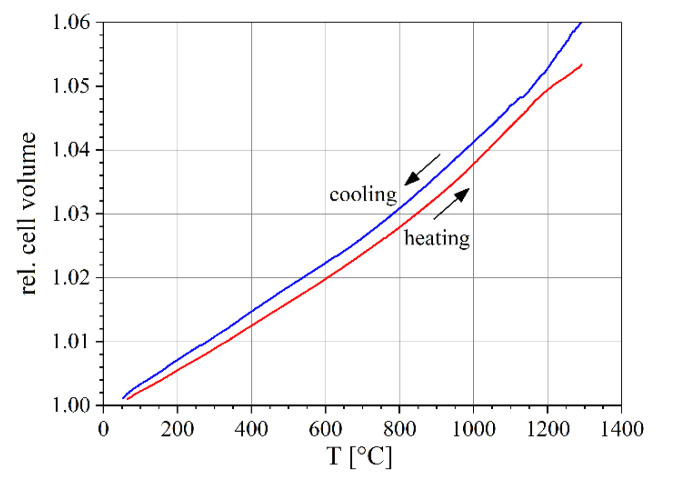
Relative mean cell volume weighted with the volume fractions of the three phases during heating and cooling (see text).

The change in the relative mean cell volumes as a function of temperature was calculated using Equation (3) (Figure 8). It combines the changes in the unit cell volume of all three phases. For better comparison, they were also normalized to their corresponding RT value. Obviously, the volume increase is not linear and there is a small hysteresis effect; the unit cell volume is slightly larger during the cooling segment.

### 3.3. Thermal Expansion Coefficients

**Figure 9:**
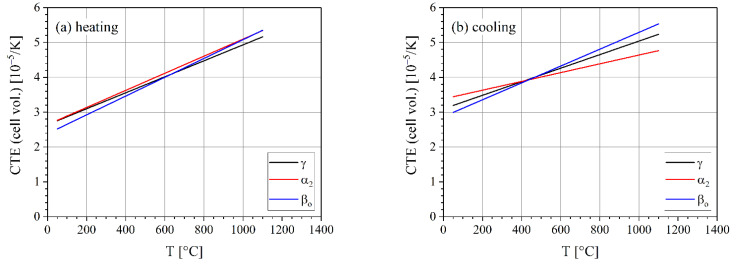
Coefficient of thermal expansion of the lattice volume for the three phases during heating (**a**) and during cooling (**b**) at a rate of 20 K min^–1^.

The temperature-dependent thermal lattice volume expansion was calculated from the cell volume for each phase by fitting a parabola to the data and calculating the derivative (cf. Equation (1)). The cell volume (i.e., volume of the unit cell) was calculated from the lattice parameters of the three crystallographic phases as described in Section 2 (Figure 9). During heating, all three phases are close together with volume expansion coefficients ranging from 2.6 × 10^–5^ K^–1^ at 50 °C to 5.2 × 10^–5^ K^–1^ at 1100 °C. During cooling, the differences are somewhat larger with α_2_ having an especially small gradient.

**Figure 10:**
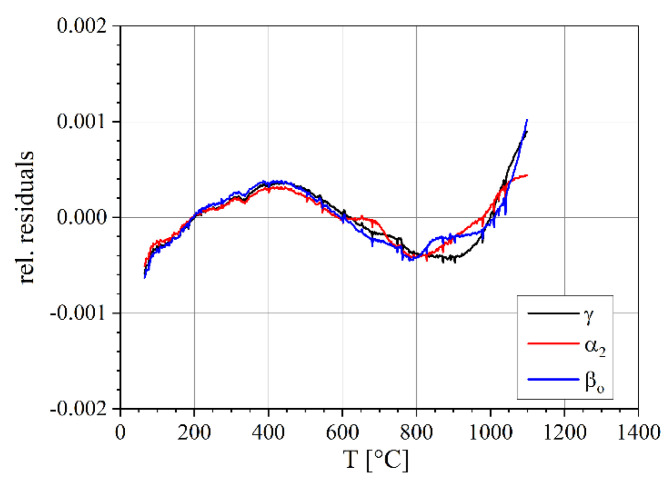
Relative residuals of fitting with a polynomial of rank 2 (heating).

The parabola fit has a relative standard deviation from the data of 3.1 × 10^–4^ (Figure 10). Thus, it describes the lattice volume expansion sufficiently well. The resulting lattice volume expansion coefficient has an error band of about ±1%.

**Figure 11:**
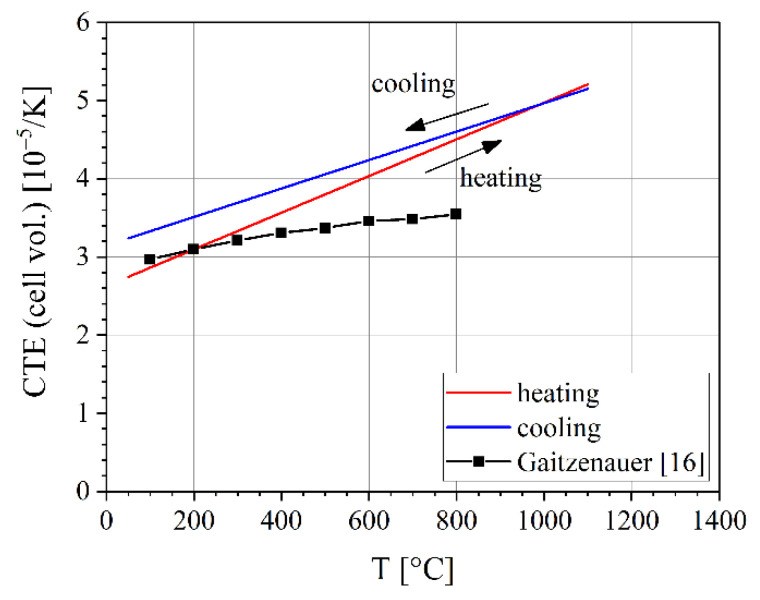
Comparison of the coefficients of thermal expansion (CTE) of the cell volume for the TNM alloy determined from the crystal lattices during heating and cooling at a rate of 20 K min^–1^. The mean value over the three phases is weighted with the volume fraction. The results of Gaitzenauer et al. [18] for the bulk are shown for comparison.

The CTE of the three phases can be combined to one CTE for the bulk material using Equation (3). The resulting CTE differ only slightly for heating and cooling, see Figure 11. For heating, the line function is CTE = 2.62 × 10^–5^ K^–1^ + 2.36 × 10^–8^ K^–2^ · *T*, for cooling, the function is CTE = 3.15 × 10^–5^ K^–1^ + 1.82 × 10^–8^ K^–2^ · *T*. A potential explanation for this difference between heating and cooling could be the different chemical compositions of the constituting phases, but more investigation is needed for a full explanation.

For a comparison with literature values, one has to keep in mind that *i*. the values can differ for different alloy compositions, *ii*. the values can differ for materials with different phase contents, and *iii*. the values determined by diffraction can differ from bulk measurements. Zhang et al. [12] give linear CTE values for three different alloys; for comparison, we calculated the volume expansion coefficient by multiplying their results with a factor of 3. The result in 10^–5^ K^–1^ is 3.3 (RT)–4.2 (600 °C) for Ti-50Al, 2.9 (200 °C)–3.5 (800 °C) for Ti-47Al-1.5(Nb,Cr,Si), and 2.6 (100 °C)–3.5 (900 °C) for Ti-47Al-4(Nb,W,B), an alloy called CTI-8.

The Handbook of Materials Data [13] reports linear CTE, again we multiplied these by a factor of 3 for comparison which gives in 10^–5^ K^–1^, 3.3 (27 °C)–3.9 (850 °C) for Ti-50Al and 2.7 (100 °C)–3.5 (700 °C) for Ti-48Al-2Cr.

Li et al. [17] report the results of heating Ti-45Al-7.5Nb-(0.25,0.5)C samples from RT to 1400 °C at a rate of 5 K min^–1^. They report a CTE value of 3.67 × 10^–5^ K^–1^ for the γ phase and 3.62 × 10^–5^ K^–1^ for the α_2_/α phases in the temperature range from 200–780 °C.

The above-mentioned literature values for different TiAl alloys (see also Table 2) fit relatively well with the results of this work, as can be seen from Figure 11, despite the fact that the alloy compositions and, thus, also the phase compositions, are different. Gaitzenauer et al. [18] determined linear CTE for a TNM alloy; their results were multiplied by a factor of three (see Equation (2)) and added to Figure 11 for comparison. While their results fit well to the current results up to about 300 °C, their CTEs are considerably lower at higher temperatures. It should be noted that it is generally expected that the bulk CTEs differ from the CTEs determined by diffraction as effects coming from vacancies or grain boundaries do not contribute to the lattice parameters.

**Figure 12:**
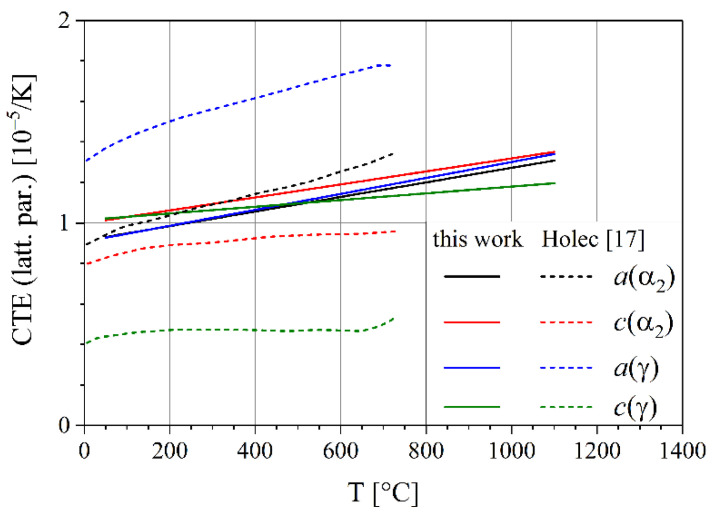
Comparison of linear coefficients of thermal expansion of the crystal lattice during heating (this work) and the work of Holec et al. [19]. The data of Holec et al., which stem from atomistic modelling, were extracted from [19].

The theoretical calculation by Holec et al. [19] fits to the current experimental data for the α_2_ phase (Figure 12). Note that in this case the linear expansion coefficient is shown, which is given in [19]. For the γ phase, *a* expands significantly faster, while *c* expands significantly slower. As a result, the volume expansion is still close to the result of this work.

### 3.4. Importance of the CTE for Internal Stresses and Cracking

Rapid cooling of materials can lead to internal stresses and cracking. However, two different cases have to be distinguished in this context: interphase stresses as well as inhomogeneous cooling.

Interphase stresses are stresses between neighboring grains that consist of different phases. The most relevant origin in this context is a different thermal expansion of the phases. In the present case, this would mostly be stresses between α_2_ and γ during cooling. As the TNM material softens around 900 °C [5], only the temperature range between RT and 900 °C is relevant for the formation of residual stresses. The strongest difference in lattice contraction (CTE) during cooling is between *c*(α) and *c*(γ), being 1.3 × 10^–3^ (relative value) from 900 °C to RT. Multiplying this with the modulus of elasticity of about 160 GPa for a rough estimate gives a stress value of 208 MPa. Consequently, cooling from temperatures above 900 °C can lead to significant internal stresses between the phases, which are termed micro-stresses or stresses of type II, but it will generally not lead to immediate cracking since this stress level is still far from the yield stress of TiAl alloys. Nevertheless, it shows that micro-stresses should in general be considered in mechanical modelling, thus requiring microstructure models at the grain level.

Inhomogeneous cooling, on the other hand, happens when a work piece is cooled from high temperatures in such a way that temperature gradients are present. Usually, this happens with work pieces of larger sizes. However, the size scale is set by the temperature gradient. This means that small samples can also develop large stresses when the temperature gradient is high. Inhomogeneous cooling leads to internal stresses between regions of different temperatures within the material. Well-known examples are welding or cooling of cast pieces. In both cases, cracks can form in the work piece that can even lead to breaking. Liu et al. [8] have shown that thin TiAl sheets can be laser beam welded as long as their size is below a few cm. With larger sizes, the sheets always break during cooling. Thus, TiAl sheets need to be welded with base metal heating prior to welding and slow cooling after welding, as also reported in [29]. The material needs to be kept above the brittle-to-ductile transition temperature during welding, which is around 700 °C, depending on the alloy composition.

The stresses that can develop in this case are much higher than the interphase stresses and occur in a single-phase material as well. The thermal strain, i.e., the relative difference in lattice parameters from 900 °C to RT is about 1% (cf. Figure 6). With a bulk modulus of elasticity of 160 GPa, this thermal strain results in a stress of about 1.6 GPa. This value is well above the yield stress, even if some hardening would be taking place, and might lead to breaking of TiAl work pieces larger than a few cm.

## 4. Summary

The coefficients of thermal expansion (CTE) of the crystal lattice were determined for a Ti-43.05Al-4.05Nb-0.94Mo-0.08B (TNM) alloy in the temperature range from 50 to 1100 °C. The knowledge about the thermal expansion of the crystal lattice is important for understanding the material behavior on the atomic scale. High-energy X-ray diffraction was used to probe the lattice parameters of all present phases in the bulk. The changes of the lattice parameters with temperature were approximated with polynomials of second order. The derivative gives linear functions for the coefficients of thermal expansion.

The results show that all three phases, i.e., γ-TiAl, α_2_-Ti_3_Al, and β_o_-TiAl, expand at similar rates during heating. During cooling, the differences between the three phases were slightly larger.

The CTE mean values were calculated over all phases with the volume fractions as weighting factors. The mean CTE increases from 2.7 to 5.2 × 10^–1^ K^–1^ during heating with 20 K min^–1^ in the temperature range from 50 to 1100 °C. The slope during cooling at the same rate was slightly different, the CTE decreased from 5.2 to 3.2 × 10^–1^ K^–1^.

The CTEs were used to estimate residual inter-phase stresses and macro-stresses during cooling, which are of importance to estimate damage that might occur during manufacturing, processing, and heating treatments.

## Figures and Tables

**Table 1:** Lattice parameters and unit cell volumes at RT for the three occurring phases as calculated with Maud. The numbers in brackets give the error in the last digit.

Phase	*a* [Å]	*c* [Å]	*V* [Å^3^]
γ	4.0160(7)	4.0560(1)	65.416
α_2_	5.7473(3)	4.6333(5)	132.540
β_o_	3.2103(1)	-	33.085

**Table 2:** CTE (volume expansion) values for various intermetallic TiAl alloys as reported in the literature. Where only the linear CTEs were given, the values were multiplied by 3 for comparison.

Author	Ref.	Alloy, Phase	T (°C)	CTE (10^–5^ K^–1^)
Zhang et al.	[12]	Ti-50Al	RT	3.3
		“	600	4.2
		Ti-47Al-1.5(Nb,Cr,Si)	200	2.9
		“	800	3.5
		Ti-47Al-4(Nb,W,B) (“CTI-8”)	100	2.6
		“	900	3.5
Handbook of materials data	[13]	Ti-50Al	27	3.3
		“	850	3.9
		Ti-48Al-2Cr	100	2.7
		“	700	3.5
Li et al.	[17]	Ti-45Al-7.5Nb-(0.25,0.5)C, γ phase	200–780	3.67
		Ti-45Al-7.5Nb-(0.25,0.5)C, αα phase	200–780	3.62
Gaitzenauer et al.	[18]	Ti-43.5Al-4Nb-1Mo-0.1B (“TNM”)	100–800	3.0–3.5
this work		Ti-43.5Al-4Nb-1Mo-0.1B (“TNM”)	50–1100	3.0–5.2 ^1^

^1^ Mean value of heating and cooling.

## Data Availability

The data presented in this study are available on request from the corresponding author. The data are not publicly available.

## References

[1] Bewlay B.P., Nag S., Suzuki A., Weimer M.J. (2016). TiAl alloys in commercial aircraft engines. Mater. High Temp..

[2] Habel U., Heutling F., Helm D., Kunze C., Smarsly W., Das G., Clemens H., Venkatesh V., Pilchak A.L., Allison J.E., Sreeramamurthy A., Boyer R., Christodoulou J., Fraser H.L., Imam M.A., Kosaka Y., Rack H.J. (2016). Forged Intermetallic γ-TiAl Based Alloy Low Pressure Turbine Blade in the Geared Turbofan. Proceedings of the 13th World Conference on Titanium.

[3] Appel F., Paul J.D.H., Oehring M. (2011). Gamma Titanium Aluminide Alloys.

[4] Clemens H., Mayer S. (2016). Intermetallic titanium aluminides in aerospace applications—Processing, microstructure and properties. Mater. High. Temp..

[5] Mayer S., Erdely P., Fischer F.D., Holec D., Kastenhuber M., Klein T., Clemens H. (2017). Intermetallic β-Solidifying γ-TiAl Based Alloys—From Fundamental Research to Application. Adv. Eng. Mat..

[6] Huber D., Werner R., Clemens H., Stockinger M. (2015). Influence of process parameter variation during thermo-mechanical processing of an intermetallic β-stabilized γ-TiAl based alloy. Mater. Charact..

[7] Schmölzer T., Liss K.-D., Zickler G.A., Watson I.J., Droessler L.M., Wallgram W., Buslaps T., Studer A., Clemens H. (2010). Phase fractions, transition and ordering temperatures in TiAl–Nb–Mo alloys: An in- and ex-situ study. Intermetallics.

[8] Liu J., Ventzke V., Staron P., Schell N., Kashaev N., Huber N. (2012). Investigation of In Situ and Conventional Post-Weld Heat Treatments on Dual-Laser-Beam-Welded γ-TiAl-Based Alloy. Adv. Eng. Mat..

[9] Kirby R.K., Maglić K.D., Cezairliyan A., Peletsky V.E. (1992). Methods of Measuring Thermal Expansion. Compendium of Thermophysical Property Measurement Methods.

[10] James J.D., Spittle J.E., Brown S.G.R., Evans R.W. (2001). A Review of Measurement Techniques for the Thermal Expansion Coefficient of Metals and Alloys at Elevated Temperatures. Meas. Sci. Technol..

[11] He Y., Schwarz R.B., Darling T., Hundley M., Whang S.H., Wang Z.M. (1997). Elastic constants and thermal expansion of single crystal γ-TiAl from 300 to 750 K. Mater. Sci. Eng. A.

[12] Zhang W.J., Reddy B.V., Deevi S.C. (2001). Physical Properties of TiAl-Base Alloys. Scripta Mater..

[13] Warlimont H., Martienssen W. (2018). Springer Handbook of Materials Data.

[14] Shull R.D., Cline J.P. (1990). High Temperature X-ray Diffractometry of Ti-Al Alloys. Materials Chemistry at High Temperatures.

[15] Yeoh L.A., Liss K.-D., Bartels A., Chladil H., Avdeev M., Clemens H., Gerling R., Buslaps T. (2007). In situ high-energy X-ray diffraction study and quantitative phase analysis in the α+ γ phase field of titanium aluminides. Scripta Mater..

[16] Liss K.-D., Funakoshi K.-I., Dippenaar R., Higo Y., Shiro A., Reid M., Suzuki H., Shobu T., Akita K. (2016). Hydrostatic compression behavior and high-pressure stabilized β-phase in γ-based titanium aluminide intermetallics. Metals.

[17] Li X., Dippenaar R., Shiro A., Shobu T., Higo Y., Reid M., Suzuki H., Akita K., Funakoshi K.I., Liss K.-D. (2018). Lattice parameter evolution during heating of Ti-45Al-7.5Nb-0.25/0.5C alloys under atmospheric and high pressures. Intermetallics.

[18] Gaitzenauer A., Schenk M., Kuchling W., Clemens H., Voigt P., Hempel R., Mayer S. (2013). Gefüge und Eigenschaften einer mehrphasigen intermetallischen Titanaluminidlegierung für innovative Leichtbauanwendungen. BHM.

[19] Holec D., Abdoshahi N., Mayer S., Clemens H. (2019). Thermal Expansion and Other Thermodynamic Properties of α2-Ti3Al and γ-TiAl Intermetallic Phases from First Principles Methods. Materials.

[20] Clemens H., Wallgram W., Kremmer S., Güther V., Otto A., Bartels A. (2008). Design of Novel β-Solidifying TiAl Alloys with Adjustable β/B2-Phase Fraction and Excellent Hot-Workability. Adv. Eng. Mater..

[21] Güther V., Allen M., Klose J., Clemens H. (2018). Metallurgical processing of titanium aluminides on industrial scale. Intermetallics.

[22] Gaitzenauer A., Müller M., Clemens H., Voigt P., Hempel R., Mayer S. (2013). Optimized Hot-forming of an Intermetallic Multi-phase γ-TiAl Based Alloy. Mater. Res. Soc. Symp. Proc..

[23] Clemens H., Mayer S. (2015). Intermetallic Titanium Aluminides as Innovative High Temperature Lightweight Structural Materials—How Materialographic Methods Have Contributed to Their Development. Pract. Metallogr..

[24] Schell N., King A., Beckmann F., Fischer T., Müller M., Schreyer A. (2014). The High Energy Materials Science Beamline (HEMS) at PETRA III. Mater. Sci. Forum.

[25] Stark A., Oehring M., Pyczak F., Schreyer A. (2011). In Situ Observation of Various Phase Transformation Paths in Nb-Rich TiAl Alloys during Quenching with Different Rates. Adv. Eng. Mater..

[26] Hammersley A.P. (1997). FIT2D: An Introduction and Overview. ESRF Internal Report ESRF97HA02T.

[27] Hammersley A.P., Svensson S.O., Hanfland M., Fitch A.N., Häusermann D. (1996). Two-Dimensional Detector Software: From Real Detector to Idealised Image or Two-Theta Scan. High Press. Res..

[28] Lutterotti L., Bortolotti M., Ischia G., Lonardelli I., Wenk H.-R. (2007). Rietveld Texture Analysis from Diffraction Images. Z. Kristallogr. Suppl..

[29] Clemens H., Glatz W., Schretter P., Klassen M., Schubert E., Sepold G., Fleischer T., Schrock H.W., Franke R. (1996). Intermetallic Gamma Titanium Aluminides—Potential Candidates for Spacecraft Structures. Proceedings of the Spacecraft Structures Materials and Mechanical Testing, ESA, SP-386.

